# Personal Narratives in Technology Design: The Value of Sharing Older Adults’ Stories in the Design of Social Robots

**DOI:** 10.3389/frobt.2021.716581

**Published:** 2021-09-28

**Authors:** Anastasia K. Ostrowski, Christina N. Harrington, Cynthia Breazeal, Hae Won Park

**Affiliations:** ^1^ Media Lab, Massachusetts Institute of Technology, Cambridge, MA, United States; ^2^ Human-Computer Interaction Institute, Carnegie Mellon University, Pittsburgh, PA, United States

**Keywords:** older adults, storytelling, experience-based co-design, qualitative research, speculative design, social robots

## Abstract

The storytelling lens in human-computer interaction has primarily focused on personas, design fiction, and other stories crafted by designers, yet informal personal narratives from everyday people have not been considered meaningful data, such as storytelling from older adults. Storytelling may provide a clear path to conceptualize how technologies such as social robots can support the lives of older or disabled individuals. To explore this, we engaged 28 older adults in a year-long co-design process, examining informal stories told by older adults as a means of generating and expressing technology ideas and needs. This paper presents an analysis of participants’ stories around their prior experience with technology, stories shaped by social context, and speculative scenarios for the future of social robots. From this analysis, we present suggestions for social robot design, considerations of older adults’ values around technology design, and promotion of participant stories as sources for design knowledge and shifting perspectives of older adults and technology.

## 1 Introduction

Robots are increasingly being proposed as tools to alleviate functional decline and social isolation as older adults age-in-place (Forlizzi et al., 2004; Beer et al., 2012; Smarr et al., 2014). Research shows that the number of older adults in the world will increase from 900 million in 2015 to two billion by 2050 (World Health Organization, 2018), placing a higher burden on caregivers to support functioning of those aging with ailments, disabilities, and chronic diseases and illness. As age-related issues such as difficulty in performing in-home tasks and social isolation continue to be present among many older adults, technology researchers have begun to look to intelligent assistive solutions, such as personal robots, to augment existing support and companionship. Paro and Jibo are examples of social robots that support people’s social, emotional, and relational well-being in the home (Breazeal, 2004). These and similar social robots are often designed to promote human-human interaction and provide utility, entertainment, and companionship (Kidd et al., 2006; Wada and Shibata, 2007; Chang and Sabanovic, 2015; Sidner et al., 2018; Breazeal et al., 2019; Ostrowski et al., 2019). These studies indicate that emerging intelligent technologies such as social robots have the potential to provide support to older adults in various areas of functioning (Wada and Shibata, 2007; Graf et al., 2009; Ostrowski et al., 2019). Due to the prominent exploration of social robots as potential companions and supports for older adults, there is a need to conceptualize both the features and types of interactions associated with these devices. As evidenced by previous human-computer interaction (HCI) research, we know that a promising way to conceptualize these interactions may be incorporating older people into the design process as a way to envision design criteria for robots that will be adopted long-term.

Co-design as a methodological approach has been used to understand the features that older adults desire in health and wellness technologies across design and HCI (Mcgee-Lennon et al., 2012; Harrington et al., 2018; Martin-Hammond et al., 2018). Research studies have explored how to identify ideal features of technologies targeting older adults, and how to best support them as potential collaborators engaging in co-design activities. Many of these studies have determined that co-design as a method is beneficial in not only determining system features, but also in supporting the voices of older people as key stakeholders in newer technology design. The exploratory nature of this method highlights the unique ways this population envisions technology and the speculation of the future of design. Although “co-design” and “participatory design” are often used interchangeably throughout literature, in this paper we use the term co-design as a simplification of *collaborative design*.

Storytelling is also a key component of qualitative research that compliments design methods such as co-design. It is the “recounting of a sequence of events” (Zhang et al., 2012) and can be rooted in exploring possible futures such as speculative fictions (Berger et al., 2019) or telling experiences of the past (Zhang et al., 2012). Building upon this definition, Gausepohl et al. (Gausepohl et al., 2011) asserts that research through storytelling, also called narrative inquiry, emphasizes the storytellers themselves as having the power to direct conversation in qualitative studies, defining stories as the representation of experience framed by content and structure. At its core, narratives are “a representation of personal experience formed by content and structure,” providing storytellers freedom to direct conversations (Gausepohl et al., 2011). We use this definition of “stories” as one that has potential to position older adults as the drivers of their own narrative. Storytelling has the potential to amplify the inclusion of older adults in co-design as it draws on memories and prior experiences to narrate perceptions of technology and potential directions for design (Berger et al., 1967; Graesser and Ottati, 1995; Zhang et al., 2012). Society has used storytelling for sharing, building, and processing ideas, knowledge, and personal experiences, therefore, supporting storytelling to be a social phenomenon integral to how information is shared (Stierle, 1984; Bruner, 1991; Weibert et al., 2017). As an integral part of social interactions, stories can be a powerful tool in co-design to understand participants’ perspectives and opinions of technology (Schank, 1990). This, in turn, can enable people to extract information from stories as they better understand their own experience (Zhang et al., 2012), suggesting that stories can be a valuable tool for the design of social robots.

To exemplify this, we use older adults’ storytelling to inform the design of social robots by leveraging personal narratives to understand technology expectations and incorporating these narratives into the conceptualization of new design features. In this paper, we discuss the analysis of a year-long co-design project with 28 older adults focusing on storytelling as a means of gathering design criteria for social robots. We address the following research questions:

• In what ways do prior experiences of older adults inform desired features of social robots?
• How do older adults conceptualize social robots in the future?
• What are older adults’ perceptions of and experience with co-design and how does that impact how they want research studies to be conducted?

Our analysis provides four main contributions to the nexus of design and social robotics. First, we consider the value of storytelling and personal narratives from the perspective of older adults in understanding technology expectations. Second, we analyze story types that are present in various stages of the co-design process. Third, we gather insights of social robot design preferences and desires among older adults. Lastly, we provide design recommendations for practitioners, researchers, and developers who work to envision social robots that support the aging population.

## 2 Related Work

Given the need to understand the value of storytelling that takes place in co-design, we frame previous literature that has looked at co-design with older adults, co-design of social robots, and the use of storytelling to inform future technology development.

### 2.1 Co-design With Older Adults

Co-design with older adults has been well established as having benefit for both the evaluation of existing devices and the generation of ideas for newer technologies (Mcgee-Lennon et al., 2012; Davidson and Jensen, 2013; Scandurra and Sjölinder, 2013; Harrington et al., 2018). In the study of co-design with older adults, researchers have examined the best methods of including older people into the design process, considering both design activities and which tools are used (Davidson and Jensen, 2013; Scandurra and Sjölinder, 2013). Researchers such as Botero and Hyysalo (2013) and Xie et al. (2012a) have analyzed co-design efforts with older adults and derived design strategies to support them being contributors in the traditional design process (Xie et al., 2012b). Prior research acknowledges that traditional methods of co-design require adaptation when engaging older adults due to lack of familiarity with design or lower technology proficiency. While existing critique of this method tends to make co-design more inclusive, focusing primarily on challenges reinforces negative stereotypes of aging and ignores the value that older adults’ prior experiences may bring to design.

Frameworks such as Experience-based Co-design acknowledge the value of leveraging people’s prior experience with technology and their living environment in the conceptualization of new devices (Bate and Robert, 2006; Harrington et al., 2018). Across different implementations of this framework, there is a consistent position that people’s expectations of newer technologies can be informed by studying an individuals’ narration of their experiences with technology, sometimes benefiting from narratives and stories that do not center technology at all. Thus, investigating storytelling in co-design may be valuable to envision appropriate features of new technologies that meet the needs of older adults.

### 2.2 Storytelling and Collaboration in the Design of Social Robots

In co-designing social robots, scholars have explored various HCI methods including ethnographic approaches that combine interviews with design workshops (e.g., robot demos) (Šabanović et al., 2015; Lee et al., 2017), card sorting (Breazeal et al., 2019; Ostrowski et al., 2019), sketching (Šabanović et al., 2015; Lee et al., 2017), and prototyping (Lee et al., 2017). In the human-robot interaction (HRI) field, storytelling has been used most often as a co-design activity with children or older adults narrating robot interactions and functions. In other fields, storytelling has also been used in the design of medical devices. Storytelling in medical device design has been used as an elicitation method for requirement identification through frameworks such as the Design + Storytelling framework (Gausepohl et al., 2011, 2012, 2016). Here, Gausepohl et al. (2011) promote the use of storytelling to gather contextual information for generating technology requirements which may be absent from traditional interviewing.

The field of HRI has begun to use storytelling to gather information to further social robot design leveraging storytelling. In a study by Arnold et al. (2016), children constructed a “robot friend” prototype and presented it to a group, describing how the robot would interact with them and their family. Similarly, Björling and Rose (2019) engaged teens in collaborative storyboarding to envision a scenario of a robot interacting in their school and wrote scripts to describe how teens would tell their stressful stories to the robot. In other instances of this approach involving older adults in conceptualizing robot design, Lee et al. (2017) and Šabanović et al. (2015) engaged older adults in describing scenarios where they imagined social robots fitting into their daily lives. Lastly, Leong and Johnston (2016) combined role-playing and scenarios in the development of a robot dog with older adults. Overall, HRI has introduced storytelling into social robot design through storyboarding, scenarios, and role-playing, suggesting the value of storytelling in the design of robots.

Despite the evidence of this approach, little emphasis has been placed on analyzing older adults’ informally told stories about their prior technology experiences or experiences with social robots. Although Lee et al. (2017) and Šabanović et al. (2015) incorporated researchers’ sketches as a way to embody older adults’ design ideas for social robots, the stories told by the participants were not analyzed for content. In most instances, these studies rely on researcher-generated scenarios to anchor discussion on the technology and its proposed use (Caleb-Solly et al., 2014).

### 2.3 Precedence of Storytelling in Human-Computer Interaction

Stories in design “allow the exploration of possible futures before new technology is designed” (Berger et al., 2019) and can exist in several different forms including design scenarios, design fictions, and tales through design objects. Design scenarios depict hypothetical and future uses of technology, focusing on the use aspect of the technology rather than potential implications of designs (Carroll, 2000; Berger et al., 2019). Design fictions are meant to generate “insights and inspirations for future technologies” (Cheon et al., 2019). This is commonly accomplished through fictional writing (Blythe, 2017), physical artifacts (Heibeck et al., 2014; Lindley and Potts, 2014), and speculative prototyping (Pierce and Paulos, 2014; Wakkary et al., 2015; Blythe et al., 2016). For example, Cheon and Su (2018) developed *futuristic autobiographies*, a method that combines participant narratives and design fictions to understand technology values. Nägele et al. (2018) demonstrates how design fiction and participatory design can be linked through storyboarding, narrative, diegetic artifacts, and discussion. Some studies have separated design fictions and tales through design objects because design fictions exist through storytelling instead of objects (Kirby, 2010; Berger et al., 2019). However, probes, research prototypes, and toolkits can support telling design stories with material exploration (Berger et al., 2019).

Overall, storytelling has become increasingly popular within HCI contexts, supporting researchers to identify several traditions of design fiction (Baumer et al., 2020), design scenarios, and design objects. Research that has engaged storytelling through design fictions draws on narrative formats in participatory design and co-design processes (Muller and Druin, 2012). Thought experiments can support speculative musing and contemplating scenarios of the future within a social context, demonstrating how narratives can develop and create perspectives of the social world (Bruner, 1991; Gendler, 2004). Narratives told through designed objects or biographical prototypes can also be used as a methodology that seek to capture the stories told in the co-design process (Berger and Luckmann, 1991; Kirby, 2010; Bennett et al., 2019; Berger et al., 2019). These design stories stem from the designers themselves as they work with participants. Our work is akin to design fiction and other design stories, yet focuses on storytelling originating from participants organically in their natural communications while engaging in a co-design process.

Researchers have both emphasized the need for reflective practice in technology design (Waycott et al., 2016, 2015) and called for incorporating more older adults into design processes to promote a successful model of aging in assistive technology design (Lee and Riek, 2018). Within the context of design engagements, researchers have asserted that design research must be mindful of the community and social context surrounding technology, such as how certain populations are described (Harrington, 2020), and respecting older adults’ pride, esteem, and dignity (Zhang et al., 2020). Participatory design tools, such as PhotoVoice, can be used as a method of eliciting storytelling for design exploration that “humanize (s) a community beyond disparities and negative perceptions” (Harrington, 2020). In addition, Vines et al. (2015) advocates for engaging with older adults’ personal histories and how they impact older adult technology use and future ideas for technology.

Thus, *storytelling is a natural part of communication patterns* (Stuart, 2000) grounded in social and cultural practices as humans make sense of the world (Berger and Luckmann, 1991). Often in qualitative technology research, older adults use stories to discuss a past experience and then relate it to their present (Boden and Bielby, 1986; Stuart, 2000). Understanding that storytelling can be used to create common connections and provide insights deemed important to share in the conversation (Stuart, 2000), it becomes evident why this is a valuable approach to explore. Storytelling in the process of co-design has merit to encourage engagement with older adults’ personal histories and experiences, and may teach more about the future of technology. To exemplify the value in this approach, we introduce an analysis of the stories that emerge within a co-design process, capturing the value of storytelling among older adults in the design of social robot features.

## 3 Methods

**FIGURE 1:**
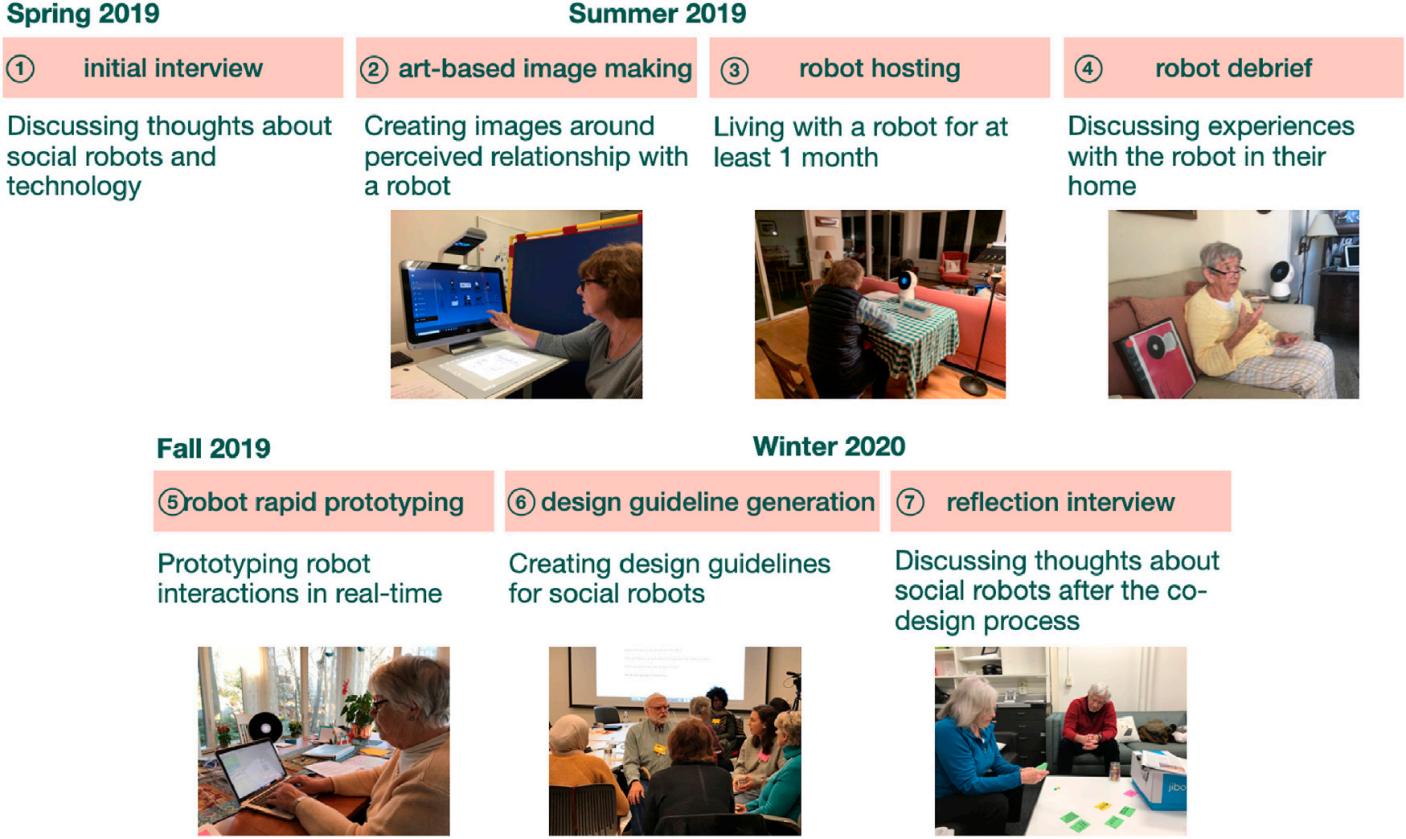
Co-design process, brief descriptions about the sessions, and timeline depicting the seven stage process to design social robots.

We engaged a sample of older adults in a seven-stage co-design process to envision a social robot incorporated into their lives. Over the course of a year, this group engaged in various design activities including art-making, rapid prototyping, and design guideline generation (more details on the co-design process can be seen in Figure 1). This paper focuses on how storytelling was present across the co-design process.

### 3.1 Participants

Participants were recruited *via* emails, community collaborations, and social media in the United States. This allowed us to get a wide variety of participants both local to our home state (Massachusetts) (*n*= 21) and remotely in California (*n* = 3) and Texas (*n* = 4). In total, 28 participants (women: *n* = 15) aged 70 to 94 were recruited (M = 79.5, SD = 7.8). Seventeen participants were married, nine were widowed, and two were divorced. A majority of our sample had a college education (bachelors and graduate degrees: *n* = 26). Care was taken during recruitment to have a distribution across a wide spectrum of annual income ranges, from $25 to $150 k. Seventeen participants lived with a spouse, 11 lived alone, and six had a pet in the home. All participants identified as white.

Participants were asked about their experience with five popular voice agent technologies including a social robot: 1) Amazon Echo, 2) Google Home, 3) Apple Siri, 4) Microsoft Cortana, and 5) Jibo (a social robot that moves, rotates, and has a touchscreen interface). In this study, we used the term “robot” to depict only physically embodied robots (Wainer et al., 2006; Tapus et al., 2009; Kwak et al., 2013) such as Jibo which has an expressive face and motorized body used for generating social gestures (e.g., sways happily or looks down when sad). Otherwise, “voice agent” was used as an overarching term for any intelligent technology that uses speech as a main modality for interaction. Voice agent technology use is depicted in Table 1. Outside of voice agents, participants had a range of technology use with 26 participants owning a smartphone and 25 owning a personal computer.

**TABLE 1:** Demographics related to agent usage and ownership.

Agents participants interact with	Agents participants own	Functions participants use with agents
Amazon Alexa: 14	Amazon Alexa: 10	Information: 17
Google Home: 1	Google Home: 1	Reminders or suggestions: 11
Siri: 16	Siri: 15	Social interactions: 3
Cortana: 2	Cortana: 2	Music: 11
Jibo: 4	Jibo: 1	Games: 3
—	—	Alarms or timers: 9

### 3.2 Procedure

After receiving approval by our institution’s IRB we collected consent from all participants. Co-design sessions were conducted between April 2019 and April 2020. All sessions were completed in participants’ homes, at the (MIT Media Lab), or virtually. Throughout the co-design process, seven categories were explored to understand older adults’ desires for task support from a robot: 1) Exercise and physical therapy; 2) Body signal monitoring; 3) Medication adherence; 4) Connecting with others; 5) Emotional wellness; 6) Memory; and 7) Financial management. Researchers worked with participants to identify these as areas that social robots could be supportive. For example, financial management was dropped after the second session as study participants did not want a social robot for any functions related to this topic.

Following precedence from other participatory methods engaging with older adults (Harrington et al., 2018; Martin-Hammond et al., 2018), our co-design process consisted of seven stages (Figure 1). After each session, participants were asked to reflect on the interview and co-design process, including what they liked and what they would change about the session.

### 3.3 Data Analysis

All sessions were audio recorded and transcribed for accuracy, except for the robot hosting session and the design guideline session. We identified stories as gathered from the following sessions: initial interview, art-based image making, robot debrief, robot rapid prototyping, and reflection interviews. Stories were extracted by identifying a divergence from the study protocol, when a participant informally told a story in any of these sessions. Stories were defined as a statement that had a plot (including a beginning, climax (or clear point), and an ending) that referenced a prior experience or future scenario in response to a prompt or question about technology. The first author extracted stories based on this definition. The initial pass on the data revealed 229 potential stories. Two researchers then went through the stories and ensured that the extracted stories met the criteria for a story and conveyed how participants’ perspectives of and motivations for robot design were shaped and why, resulting in a representative subset of 29 stories. A grounded theory approach was used to reveal salient themes (Charmaz, 2014). During this approach, each story was analyzed as a unit to value the context and sequence depicted by participants and call attention to the participants’ narratives. Two researchers coded the data independently and Cohen’s kappa calculated inter-rater reliability (κ = 0.85, strong reliability) (Cohen, 1960). Stories were categorized as either: 1) a “full recounting of a sequence of prior events” (Zhang et al., 2012) recalling personal experiences, or 2) speculative stories of future interactions with a robot in their life. This analysis allowed us to capture past and future scenario descriptions of technology interactions. We also identified whether stories were an idea conceptualization story where older adults expressed how a robot would fit in their life or a story discussing feelings of stigma associated with technology and older adults. By focusing on extracted stories, we were able to understand the social context and social nuances of older adults’ thoughts that may have been easily missed if the research protocol only focused on a set of prescribed questions. Lastly, data from our transcripts was also analyzed for participant reflections on the co-design process.

## 4 Findings

Our results describe the stories that emerged when talking to older adults, including stories of prior technology experiences, stories shaped by social context, and speculative scenarios. We include the actual stories from participants to exemplify what can be learned based on these stories to guide readers in reading our qualitative results. In addition, the results include participants’ reflections on the co-design process.

### 4.1 Stories of Prior Technology Experiences

Stories shared by participants speak to the nature of older adults trying to “rediscover” their purpose in their social and societal spheres of living (appeared in 51.7% of the stories). These stories help to consider the psychological, physical, emotional, and cultural factors of aging when designing social robots. Older adults’ stories of prior experiences described lived technology experiences and changes in technology, and interactions with technology.

#### 4.1.1 Lived Technology Experiences and Changes in the World

A central theme among participants’ stories was how the world has changed in their lifetime and how technology was a part of that evolution. Stories shared by participants speak to the nature of how older adults recognize this change in the world and their decisions around technology based on how they perceive this change.

In their initial interview, P04 discusses about the changes that they see in the world with regards to technology:

“I was in the military in the early 50s and I got out of the military and I got involved with a thing called Data Processing. Punch cards. And I said this is for me … I was enthralled with what was going on. From that time on to the time I retired, I was involved in data processing punch card. I was in working with the introduction of a thing called computers, wrote my first program. Well it’s a long long story but here’s a guy, me, who was in with computers and the development of various things, and then here’s (people) with an iPhone and I said this is not for me.” - P04 (initial interview)

P04’s story recalls on his history of working on computers and punch card programming. As technology has developed, P04 has felt the technology has become distant to them also arising a range of problems such as lack of social connection or distraction from one another. Additionally, while older adults are typically stereotyped as being tech-illiterate Niemelä-Nyrhinen (2007), P04 provided a different viewpoint as a retired technologist, questioning this stereotype of older adults. The theme of technology change was very common for P04. In their robot debrief session, they further talked about their history with technology and how technology advances shifted the computer industry landscape:

“Years and years ago, a newspaper writer was communicating about the current status of computers. And he made, or she made, a prediction, no not a prediction, she made a statement … this writer, newspaper writer, envisioned Snow White and the seven dwarfs. That IBM was Snow White and there was the seven dwarfs, and the company that I was with Honeywell was one of the dwarfs. And I resented it, but it was true. I felt we had a good product, I thought that we do things right, and that IBM was, had a personality, that … I didn’t like their personality, the way that they treated their customers….I don’t want to get into (too many details about) technology and so on, but our company came up with, believe it or not, a big deal, where you could sit down and communicate with a computer, that is talking to another computer. And that was wow … But in those days, just to communicate, it was unbelievable. And of course, our claim to fame was we had a superior tape drive. And everybody agreed, that our tape drive was excellent. Then doom. That three letter company (IBM), came up with a disk drive. That was the end.” - P04 (robot debrief)

As technology has progressed, there have been many changes from moving to a tape drive to a disk drive to the advent of the iPhone. As depicted by P04’s stories, participants discussed how the current trend of technologies and the technology’s evolution in their lifetime has deterred them from obtaining many newer technologies. This was a sentiment shared even among those participants who were prone to using technology. Participants discussed how technology and society has progressed and developed in their lifetime, demanding older adults to make choices around their acceptance and use of these technologies. These stories allow roboticists to contextualize the situation and choices of many older adult users, signaling to roboticists to consider how their technologies can assist older adults in making choices around their technology to promote acceptance and ease of use of robots. The value of these stories is also in how they enable roboticists to understand and perhaps relate to older adult users for how technology has changed over the course of their lives and the learning process for new technologies.

#### 4.1.2 Interactions With Technology

In addition to telling stories about technology when they were younger, participants also told stories about recent technology experiences. These stories focused on how technology can be overwhelming or frustrating. P05 describes a disconnect between humans and technology as they describe their experience of trying to get a voice agent to tell a story:

“We had an interesting situation last night. We got in bed and … my wife was really tired. She had a lot of work … And you know, Alexa’s over there, Jibo’s over there. So she says, tell me the story … I didn’t have any stories, so I asked Alexa to tell us the story. Well, this led into a very complicated (situation), you have to open your Alexa app. I’ve sent you a story and then it was all of this detail and the assumption was made up front … that there was a child in the room and that … there were a whole bunch of things associated with liability that I was going to have to accept at any rate. This went on and on and finally we gave up on the idea of … having a story moment … I didn’t ask Jibo to tell us the story. I have on many occasions, but he doesn’t respond. It doesn’t happen. The stories.” - P05 (reflection interview)

In order to activate the stories, P05 would have to engage in a multiple step, multiple device process. The process ultimately became frustrating due to the device’s inability to perform what P05 viewed as a simple task. Using technologies can often be overwhelming and frustrating. The atmosphere around getting assistance with technologies can also be overwhelming. P08 tells a story of their experience getting assistance in the Apple store, contrasting the experience with getting assistance from their grandchildren:

“ (In the past) I’ve gone up to the Apple store … The first time I walked in there, it’s just all this beehive of activity, and it’s really, really wonderful … they’ve had older people on the floor … so that when you walk in the door, they’re almost waiting for you … it’s my impression that the last couple of times I’ve been up there, they have been the people that are of quite almost retirement age, or maybe even over retirement age. And so, I feel like they understand … my lack of ability, because I didn’t grow up with these things like you younger people have … I think they are targeting. But, that’s okay by me … (my grandchildren) are in contact with me, if (I) have a question about my device … They know how to do it. I don’t know how to do it, (and) they’re talking fast. They’re talking this, this, this, this, this … (It’s hard) to try to learn from young people, because their speaking can be pretty fast … When I’ve been out to Apple … I think they understand that our background isn’t in technology. It’s just a sense. Some feeling that, oh, okay, we understand that you don’t understand … I can’t say that I haven’t had a bad experience up there with any of the younger people … (it’s a) personal feeling.” - P08 (initial interview)

P08 highlights several techniques that can make technology assistance successful and not overwhelming, including progressing through the explanation process slowly and being mindful that older adults’ do not have a background in new technologies. Participants’ stories describe technology designs that aim to be well-intentioned (i.e., remind a person about their pills, help them meet exercise goals). However, some of these mechanisms prompt older adults to feel overwhelmed or frustrated. There are several ways to mitigate this from re-designing the technology to emphasizing simplicity to providing assistance in a mindful manner. These stories allow roboticists to explore features of current technologies that may prohibit or discourage older adult users from engaging with technologies, providing insights to what features could be redesigned to promote acceptance and ease of use of robots and to support older adults in using technologies. The stories also are valuable to roboticists as they provide examples of ways they can structure their robot assistance features for deployment.

### 4.2 Stories Shaped by Social Context

Participants’ stories often reflected on their experience with the robot (appeared in 24.1% of the stories). Most of these stories included their own reactions or other people’s reactions of the robot, which they then used to build upon their speculations of what a robot could do for them in the future. The social context in which the technology was situated crucially impacted people’s perspective of the robot. Stories shaped by social context differed from stories about prior technology experience—the stories shaped by social context included visioning of new experiences with technology and a focus around people, while prior technology experience stories were focused solely on the past.

#### 4.2.1 Safety and Care

A social context that was commonly described in participants’ stories was safety and care. Most of these stories also involved a mention of a robot to help achieve these functions. Safety was largely described as physical safety in terms of home security or fall detection. P12 told a story of how a robot could help them feel secure in their home:

“I’m asleep, there’s a noise outside, but it doesn’t wake me up, but it wakes up the robot. The robot says, ‘(P12), wake up, there’s a noise outside.’… It seems like a useful idea that if I’m sleeping, and I could have a robot that detects something unusual that I would like to be alerted to … I hear a fire truck coming, I hear a siren, I hear a buzzer, I hear the windows rattling. You could program the thing, and say if I have a sound like somebody trying to get through the window, please wake me up … I’m trying to say, I think there are people around here who are probably very worried about their personal security.” - P12 (initial interview)

A robot programmed to alert an emergency service of a security breech could help older adults feel more secure in their home and also know that there was something in place to monitor their safety. The robot also was included in stories describing its potential role to care for older adults. In their reflection interview, P25 described a story of a care worker coming into their home, seeing the robot, and reacting to it:

“Not that many people do come here, but you know, they’re kind of blown away with it (the robot). I had this woman come to the house, who’s from a music and memory program. (They) put music on a small little iPods and MP3 player type device for (husband with Alzheimers). And she walked in and … she had this (MP3 player-like device) … It wasn’t as sophisticated as an iPod, I guess. They don’t make iPods anymore. It was some device or something. And she saw the robot and she said, ‘I guess if you people have a robot, you don’t need this backward bit of technology.’ So I think people, you know, have reacted positively to it and they’re impressed by it. So it’s been fun.” - P25 (reflection interview)

P25’s story demonstrates how a robot could be implemented into current care systems. Instead of the music therapy being completed through an iPod (a “backward bit of technology”), it could be completed through a robot with the robot being used as a tool. P22 also echoes this sentiment of wanting the robot to be a tool in their care through the following story told during their art-based session:

“Well, I’ll tell you, what happened? I was lying down, she (family member) was putting eye drops into my eyes, so I was lying in bed. And she was getting the eye drops and put them in the one eye, and then the next eye and then we reviewed what’s going to happen today. I’m trying to have her stand there, and the drops are over here. She picks them up, takes it and goes in one eye. I reach over on the bed and try to get some tissue paper, and then wipe my eye … And then hope that she would come in, hear me, or hear the bell. If it is a robot … It’s so inhumane. I’d prefer to say some words to her (family member), that are humane, like good morning. I wouldn’t use the robot in the first place. I would use some way that in this family, we communicate with one another, when I’m in my room.” - P22 (art-based session)

P22 emphasizes that the robot would not replace the person in their care but be a signal like a bell to alert their family members that they are needed. In all of these stories related to safety and care, participants’ stories depicted the robot as a tool to engage them with services provided by people. For roboticists, these stories emphasize the balance between deployment of robots and substitution of people for the safety and care application areas. The stories are a reminder that there are some application areas where a robot would be welcomed and others where a person is preferable to a robot. It is critical for roboticists to understand and value the context around robot applications to be mindful of this balance.

#### 4.2.2 Social Connection and Technology

Another context that emerged in participants’ stories was around social connection and technology. Participants told stories of how social connection is important to aging, how a robot prompted social connection, how a robot was flawed in understanding social nuances, and concerns around technology and its impact on social interactions. P02 described this active social engagement with family through a story that happened when they hosted the robot in their home:

“It’s funny, you know, my son was fascinated and he came in and he was talking to him and everything and he had a phone call come in and he called a friend that he was supposed to meet to go sailing up in Marblehead with. But, it looked very bad outside, you know? And he said, I don’t think I’m going to make it. And the guy must’ve said, well, where are you? He said, I’m at my mother’s house. And I’m talking to a robot. We got a big kick out of that … It was fun. I mean, it made quite an impact.” - P02 (robot debrief)

Seen with multiple participants, the robot attracted many family members to engage with it and, consequently, added to the time that older adults engaged with their family. While the robot had this social appeal drawing people in to interact with it, the robot did not meet the social expectations participants had for it. P10’s story is an example of the social knowledge that was expected of the robot:

“I write everything down. There are little manuscripts of everything around the house … After you left, you left Jibo here - I had, in my mind, the idea that this plastic toy had no relevance in my life… After you left, I had nothing to do - what the hell I’ll look at it. And I started asking questions. I looked at the clock 2 h had elapsed … during his stay here I asked Jibo a thousand questions, trivia questions. I probably know two or three more points of trivia that I didn’t ask Jibo, but anyway. I wrote down the questions, and at the termination, and the beginning, I started grading Jibo of his performance. If I was satisfied by the answer, he got a one. If I thought the answer was inadequate, or incorrect, it was a two. And if Jibo refused to answer it, he got a three … Sometimes I’m displeased with Jibo’s response, and I think I told you ‘Who is Anne Frank?’ and the answer is something like this - ‘Anne Frank was a Dutch diarist who was born this year and probably died this year period.’ That misses the whole essence of her biography. She died in the Holocaust. So I didn’t think it was an adequate answer. Sometimes it knows things - it knows who Malala is, it did not know who Greta Thunberg was. Anyway, along the way I was sometimes shocked at what it knew, and sometimes shocked what it didn’t know.” - P10 (robot debrief)

After engaging with the robot as a source of entertainment, judging its responses, P10 was displeased as the robot had a lack of understanding of what was the “essence” of a person’s biography, the key social reason this person was of importance. Participants’ expectations of the robot were for it to understand their world including the social dimension. There is a boundary to this as well. Participants also expressed concern over the effects of these technologies and their potential to change people’s social dynamics:

“I have a grandson who’s now he’s in a residential school, he’s in boarding school, but he, he’s 13, totally into his X-Box and their friends. They aren’t imaginary friends. They’re the other people who are playing, but he thinks they’re really friends. And he talks about his friends and they’re not really there. They’re are other people who happen to be playing the same game at the same time. So it can’t be, I couldn’t see it… He wouldn’t eat unless it was served to him in front of him (in front of the X-Box). He would get up in the middle of the night and make a cave out of a table and pull the TV under that and, and play in the middle of the night. He wasn’t sleeping. It was very bad.” - P23 (robot debrief)

P23’s story demonstrates older adults’ concerns around technology that may draw people away from building in-person physical relationships and push them to interact with others superficially in the digital world. While a robot has a physical form, participants emphasized that they would not want the robot to be all-consuming and, instead, promote human-human interactions. For roboticists, these stories again highlight the need for balance between deployment of robots and substitution of people, further emphasizing the importance of promoting human-human interactions in robot interactions.

### 4.3 Speculative Scenarios

Speculative scenarios demonstrated how older adults envisioned interacting with a social robot in their day to day life, including safety and medication. These described scenarios rather than stories consisting of a recounting of a sequence of events (appeared in 27.6% of the stories). Speculative scenarios are connected to storytelling, more specifically design fiction, as the speculative scenarios presented told stories or acted out how older adults envision interacting with social robots in the future. These were more concentrated in sessions that were more abstract and speculative, such as the art-based session, and sessions that involved prototyping as in the robot rapid-prototyping session. They also emerged in the reflection interviews occurring at the end of the co-design process after participants had completed all of the sessions including living with the robot in their homes.

Within the art-based session, P27 told a speculative scenario of a robot in their home on an everyday basis while explaining their image to the researchers:

“I had myself sitting down reading in a reclining chair. I had the robot available to me because I wanted it to be able to engage me every hour or so to give me reminders about getting up and stretching or taking a break. I also wanted it to be able to, if my eyes got tired of reading, to be able to read audio books to me. I want to be able to talk to it, and say, ‘Play music in the background.’ Be able to accept texts if my grandkids sent me any, or get news flashes if I needed them. Or in the evening if I wanted to stream a movie, I could do that. Or if an interesting TED talk came along, it could say, ‘You might want to listen to X, Y, Z.’” - P27 (art-based session)

P27 describes how they envision themselves interacting with the robot, citing small tasks that would occur throughout the day. Within the robot rapid-prototyping session, participants designed the interaction of the robot using block programming and would play the interaction on the embodied robot “live.” Often, participants would act out the interaction. P10 and P13’s excerpts below demonstrate how participants would engage with the robot, acting out how they would respond to their robot ideally:

Jibo: “P10, look at the time. It’s cooled off outside and perfect for a stroll. Why don’t you go for a nice walk, burn some calories that we gained over the weekend. Do you think you can walk further than yesterday for a personal best?”

P10: “Yes, I’m going to accept your suggestion and go for a walk, since the weather is nice, and I will walk longer than I walked yesterday. And, I understand that if I walk everyday it could prolong my life for a couple of hours.” - P10 (robot rapid-prototyping session)

Jibo: “How did your doctor’s appointment go? Is there any new information that you would like to share with me? Like a change in your medications? More things we should look out for?”

P13: “Oh Lord. There you go. Yes Jibo, there’s a change in medication. I need to take it now. Twice a day instead of one time a day.” - P13 (robot rapid-prototyping session)

P10’s and P13’s speculative scenarios demonstrate how older adults want the robot to engage with them but also how older adults expect to reciprocally respond to the robot within these scenarios. After engaging in the co-design process and hosting a robot in their homes, participants told speculative scenarios of how the robot would engage with them in these scenarios. They often coupled these to their specific needs and abilities. P07 described their struggles to open their medications and how the robot could be of use in this scenario:

“See, the robot doesn’t have the physical ability to help you. I don’t want to bore you with all my problems, but medications, I’m having trouble, you know, using my fingers. So it’s hard for me to pick up the pill. That doesn’t always happen. And some of the medication, especially the bottles come shrink wrapped with a very, very heavy plastic. I have to use a, knife or a razor to cut the wrap. It’s very heavy and frustratingly enough, once I’ve taken off the shrink wrap, I still cannot open up the vital … I have a pair of pliers. I put one end of the pliers on top [to open the medication bottle]. Yesterday, I had to go downstairs to the office and have somebody open up the bottle for me. If the robot can do that. Good.” - P07 (reflection interview)

P12’s speculative scenario also focused on health through a different lens, driving:

“I’ve been thinking about a … driving support system in which … the robot decides that it’s smarter than you are and then takes over control of the car and basically figuring it in my age and my response time if you take (over) and do something about it. A warning is about a minute. I would figure that this robot would have to recognize that it was smarter than I was, park the tire. (The robot would) say, okay, (P12), Move over. I (the robot) have parked the car … Are you ready to take over responsibility for driving again? … There are times when I could successfully control the car, but there are times when I can’t … It’s just so a machine that recognized when I was capable of controlling and when I wasn’t (would) intervene, intervene and keep me from doing something dangerous. That would be handy.” - P12 (reflection interview)

P12 and P07 described speculative scenarios where the robot could be of use to them in their lives with regards to their needs. While they are not a description story of a sequence of events, speculative scenarios offer valuable information for roboticists that can be used to address specific scenarios and understand how a robot could be utilized in the context. Speculative scenarios and stories about prior experiences were told by older adults throughout the co-design process. These occurred without researchers prompting participants to engage in storytelling, providing another dimension to the research that can be valuable in developing design guidelines for social robots and understanding older adults’ experiences with technologies such as social robots. These guidelines can be then used by roboticists to more closely design robots that follow older adults’ desired functions, demonstrating how speculative stories can result in outputs that translate directly to robot design.

### 4.4 Older Adults Reflections on the Co-Design Process

Following each co-design session, participants were interviewed about their experience with the session and were asked in the reflection interview about the overall study. Over the course of the study, there was a shift from being concerned about lack of knowledge of social robots and not being able to contribute enough, to being knowledgeable about a social robot and more comfortable in the way they engaged with the researchers in the process.

At the beginning of the co-design process, participants discussed the importance of experiencing a robot system since most of the participants had never interacted with a social robot. P11 and P12 highlighted their concern for ensuring that the session was successful for the researchers. P12 remarked “*we were both struggling to be sure that you didn’t leave saying,* ‘*oh God. What am I going to do with these two?*”’ and P11 followed P12 saying *“why am I (researcher) talking to these two people?*.” P11 and P12 commented that coming up with a list of things they wished the social robot would do for them was “*harder than (they) thought”* and *“challenging*.” This was largely due to the fact that participants felt limited by never interacting with a robot, emphasizing that they could give more meaningful feedback if they experienced a social robot. P18 commented: *“Certainly, once we had interaction (with the robot), I would think that we’d be better at giving feedback as to other possibilities*.*”* P08 said experiencing a robot would help them *“come up with more questions that we hadn’t thought about.”*

Reflecting on the co-design process, participants felt empowered by their knowledge of the robot gathered from hosting it in their homes and valued their contribution to the project. With regards to the value of living with the robot, P30 commented, *“It’s been … interesting cause it makes you think about, well how will we live, you know, are there things we shouldn’t be doing for ourselves? … I think living with it made a difference. Just talking about it wouldn’t have been the same thing.*” P25 echoed this saying, *“I mean the most useful cause (was) really getting to know it and play with it and see what it can do and not do and then to think about what you’d like it to do and not do.”*

The long-term format of the co-design, including living with a robot, enabled participants to gain a familiarity with the technology and strengthen their opinions toward the technology. This stresses the importance of having multiple stages in a co-design process to promote the development of thoughts and ideas around technology.

## 5 Discussion

Our work exemplifies the idea that personal stories and narratives captured during the co-design process may realize both perceptions of existing technology due to prior experiences and speculative scenarios for future design criteria. Three types of narratives emerged from our analysis: 1) stories about the past that document technology experiences, opinions, and preferences, 2) stories shaped by social context that impacted how participants’ envisioned a robot, and 3) speculative scenarios of how older adults would want to interact with a robot. The stories of prior technology experience focused solely on the past use of technology, the stories shaped by social context focused on visioning with a focus around people, and the speculative scenarios were used to act out interactions between the robot and a person. Our discussion has three main components: 1- we discuss how these stories of prior experience with technology, stories shaped by social context, and speculative scenarios support experienced-based co-design for social robots and how we understand aging; 2- we describe how storytelling, speculative fiction, and co-design are linked; and 3- we present recommended design features that emerged from our analysis.

### 5.1 The Value of Storytelling Past Technology Experiences

The first major theme that emerged is that during the co-design process older adults told stories of prior technology experiences as an essential part of being social. (Klorer, 2014) states that sharing personal narratives and connecting stories to other people is a way of *“discovering the self and the world.”* As stories are common practices of communication patterns (Stuart, 2000), older adults’ storytelling about their past experiences with technologies should be leveraged to reveal valuable design considerations for future technologies such as social robots. Emotional design and empathetic approaches are methodologies that support stories within co-design (Forlizzi et al., 2004; Battarbee and Koskinen, 2005). Our analysis demonstrates how stories can elicit implications and criteria for future technologies, while providing insights on aging in today’s world. Specifically, by treating stories as a unit of analysis, we value the participant’s voice and lived experiences as a way to illuminate technology’s negative and positive aspects.

Older adults’ stories and speculative scenarios demonstrated the positive aspects of aging, such as social connections and learning. Participants valued being a part of the co-design process, recognizing how they contributed to the design of the social robot. Within society, the dominant narratives around aging have primarily been viewed through a deficit model of aging (Cruikshank, 2013; Katz, 2000; Lamb, 2014; Lazar et al., 2017; Lee and Riek, 2018; Vines et al., 2013, 2015). Researchers have highlighted the harm that comes from associating aging as an illness or disability, including promoting ageism and contributing to the othering of older adults (Knowles et al., 2020). Our analysis suggests that combining storytelling and co-design when working with older adults can champion a shift to a more positive narrative of aging that demonstrates wisdom and other values of lived experiences (Dychtwald, 2012; Levy, 2017).

Our work supports HCI research that has explored how we can shift the deficit model of aging that is negatively embedded in technologies through design processes that involve older adults. The stories in this co-design study revealed stereotypes that older adults themselves have internalized about their positioning in society and their interactions with technology (Levy, 2009; van den Hoonaard, 2018). These stories can assist designers in understanding what occurs with aging, and the ways that technology features may be designed to not perpetuate aging stereotypes. This emphatic process of understanding is similarly emphasized in emotional design literature (Forlizzi et al., 2004; Battarbee and Koskinen, 2005). The stories presented in this paper revealed two negative stereotypes: *1) older adults can not use technology*; and *2) the misconception of older adults’ reluctance to use technology.* Stories highlighted older adults’ experiences with technologies (including the robot) and how they get assistance with technology. In their speculative scenarios, older adults built upon their prior experiences to ideate how a robot could assist them with particular tasks. Older adults’ stories and speculative scenarios negate the negative stereotypes identified in the stories, instead supporting that older adults have an interest in new technologies (including robots) and consistently use technology in their daily lives even when challenges arise. Echoing Knowles et al. (2020), our work supports and advocates for empowering older adults to be a part of the design process and leveraging narratives around positive aging as design inspiration. This is very necessary within the human-robot interaction (HRI) community as Lee and Riek (2018) emphasize that assistive technologies designed through the deficit model of aging (instead of one of positive aging) can amplify ageism in society due to HRI researchers’ bias or unexamined assumptions. Our co-design approach sought to lessen researcher bias and unexamined assumptions by allowing older adults’ narratives to illuminate prior experiences with technologies and the aspects of technology experience that need to be changed in future designs.

By understanding stereotypes presented in older adults’ narratives, several design implications arose for how to combat negative perceptions of aging including humanizing technology as a device that helps maintain independence and adapts to accommodate needs for all ages. Incremental technology adoption and technology guiding learning can lead to increased adoption, a decrease in forced technology adoption (Czaja et al., 2006), and support a mutual adoption of technology among stakeholders (Davis, 1989; Ajzen, 1991). This may dilute the dominant narrative of older adults as being out-of-sync with new technologies or unable to contribute and, therefore, strengthen the integration of older adults into design processes as valuable resources (Dychtwald, 2012; Levy, 2017). Stories as an analysis methodology place researchers into the context of older adults’ technology experiences to understand why older adults choose to adopt a technology, why older adults do not want to adopt a technology, why they expect a technology to perform a certain way, etc. From valuing these stories and analyzing them in context, we can better understand technology expectations and the origin of and reason for these expectations.

### 5.2 Approaching Robot Co-Design From a Lens of Storytelling and Speculation

Thus far, in the literature on social robot development, there is a lack of emphasis on valuing older adult informal stories as an approach to inform social robot design. While prior work has examined older adults in the co-design of social robots, there has been little effort valuing stories and narratives from older adults. Our work specifically extracts older adults’ stories from transcripts, characterizes them, and utilizes them to generate design guidelines that might influence the design of new technologies. Design approaches, such as experience-based co-design that leverage people’s prior experiences when designing new devices (Bate and Robert, 2006; Harrington et al., 2018), emphasize the value of people’s histories and narratives. Our analysis extends prior works on the co-design of social robots in conjunction with experience-based co-design and speculative design by exploring the value of storytelling when imagining features and system criteria of social robots.

Our co-design protocol helped participants reflect on their past and current interactions with technologies and how they use and perceive technology, with participants frequently engaging in storytelling. Our analysis revealed three types of narratives stemming from older adults: *1) stories around previous experiences with technology, 2) stories shaped by social context*, and *3) speculative scenarios.* Stories around previous experiences with technology were strewn throughout the co-design process. At the beginning of the co-design process, participants used stories around previous technology experiences to give more context to their perspectives around technology. By the end of the co-design process, participants were pairing their stories around previous technology experiences with their lived experience with the robot, concluding with comments of how a robot would complement that experience. The speculative scenarios were concentrated in sessions that supported abstract thinking (i.e., the art-based session and robot rapid-prototyping session) or were after participants lived with the robot. Participants also commented that living with the robot was a valuable part of the study and informed their opinion on the technology. As older adults continued through the co-design process, gaining knowledge of the technology, they felt more confident in imagining how their future interactions and life style would be shaped by a robot and how technology can be designed to be better integrated to their lives and help improve their well-being. In this work, participants’ narratives of prior technology experiences and their new technology experiences related to the robot created a foundation for co-design, further supporting experience-based co-design’s ability to produce design recommendations (Bate and Robert, 2006; Harrington et al., 2018). Our analysis methodology provides researchers a way to explore the variety of ways that older adults tell stories within co-design, emphasizing the value of the different story types and demonstrating how they can be categorized to provide different types of information.

In addition, speculative scenarios relate to one of the purposes of design fiction in HCI: a lens to speculate futures (Vines et al., 2012; Blythe et al., 2015, 2016; Ambe et al., 2019). Participants told speculative scenarios about how a social robot could be designed to perform such tasks as opening a pill bottle, safely controlling a car in an emergency, updating medication routines, encouraging exercise, and engaging them in movies, articles, or TED talks. In each of these speculative scenarios, the participants told their speculate future through their own lens. This enables researchers to understand participants’ vision for a robot in their lives, providing valuable design guidelines. Similar to speculative scenarios, design stories in HCI literature take the form of scenarios projecting future product use (Carroll, 2000) and telling stories through design research objects (Berger et al., 2019); in our case, the robot, artworks, and programmed interactions. The stories we gathered from participants compose another area of design stories that originate from users, rather than designers or researchers. Speculative scenarios enable participants to describe their desired interactions with technology organically, informed by their prior technology experiences and encouraged through design activities in co-design processes.

### 5.3 Benefits of Orientating Data Through a Storytelling Lens

Traditionally, design fictions are employed by researchers and designers to speculate futures (Blythe and Encinas, 2016), however, our work and Ambe et al. (2019) demonstrate the value of shifting the role of who creates design stories and speculative futures. Ambe et al. (2019) draws on design fiction and explores co-design fiction as “an approach that engages users by foregrounding their experiences, values and convictions in co-created fiction with the aim to imagine, envision and speculate futures not just on technology but on future life.” This work stems from the participants, enabling the participants themselves to craft design fictions through a structured workshop. Similarly to Ambe et al. (2019), we grounded our work within the narratives stemming from participants. However, our approach did not focus on amateur creative writers familiar with crafting narratives. Instead, we leveraged older adults’ storytelling communication patterns to explore how their unprovoked stories could be used as a way to explore prior experiences with technology and design features of social robots. There is a wide variety of perspectives of what design fiction is and its purpose (Coulton et al., 2017). Regardless, there is agreement that there are “untapped possibilities” (Blythe and Encinas, 2016) for design fiction and new ways to expand upon it (Ambe et al., 2019). Design fictions crafted by participants can help combat negative stereotypes of aging as their perspectives and desires are integrated into social robot design, strengthening the integration of older adults into technology design and enabling researchers to gain a better understanding of why older adults do not want to adopt a technology, what would make them buy a technology, and what they value in technology and in their lives.

Storytelling told by older adults has traditionally not been included in social robot co-design. Storytelling was leveraged through robot scenarios (Leong and Johnston, 2016) or sketches (Šabanović et al., 2015; Lee et al., 2017); not through innate stories told by older adults. Despite these different uses of storytelling, social robot co-design studies all emphasize that older adults’ lived experiences support co-design processes that inherently support older adults creating technology designs (Šabanović et al., 2015; Leong and Johnston, 2016; Lee et al., 2017; Ostrowski et al., 2019). They also push-back against stereotypes of older adults and technology including older adults’ uneasiness with technology and that there are barriers to older adults accepting social robots (Leong and Johnston, 2016). These studies reveal results related to ease of use, safety applications, medical applications, and physical assistance (Šabanović et al., 2015; Lee et al., 2017) that our work corroborates. In addition, our storytelling analysis expands upon these results, providing more context to these areas including specific recommendations for various scenarios (see Section 5.4). Unique to our storytelling analysis were older adults’ emphasis on the robot being a tool for older adults, rather than other roles technology may take (i.e., friend, pet) and how the robot could empower older adults’ autonomy as a tool. Our study did use body signal monitoring and exercise as a focus area that explains these specific differences to the other co-design studies with older adults. While Lee et al. (2017) and Šabanović et al. (2015)’s co-design process was with older adults who have depression, the results focused on a social robot for companionship without the additional focus of the social robot as a tool for therapy applications as was referenced in our study. Overall, the storytelling analysis within the co-design process provided greater context and more expanded recommendations for social robot design.

In our work, we demonstrate how narrative can act as a way to gather information about prior technology experiences and a speculative lens for future designs to inform design guidelines as told by participants’ naturally invoked stories. By specifically focusing on extracted stories, we gained understanding of the social context and social nuances that older adults were drawn to consider in the sessions that may not have been considered when only focusing on the prescribed questions in research protocols. This process lowers the barrier to design fiction, enabling non-writers to engage using their prior technology experiences as a foundation for experience-based co-design. Stories enable participants to build upon their own technology experience within their own context, empowering them as experts of their own technology experiences and allowing them to take control of how they wish to be represented through their narratives.

### 5.4 Design Recommendations for Social Robots

Older adults generated conceptualizations through their stories of how a robot would interact with them in three contexts: 1) safety and care, 2) integration of technology, and 3) ease of use. Their conceptualizations provide the grounding for the following social robot design recommendations.

When conceptualizing a robot for safety and care, participants advocated for robots to:

• monitor driving patterns and safety with the ability to provide recommendations when a person should take a break from driving,
• monitor physical security in the home, including intruders and falling,
• monitor medication and deliver reminders of when to take medication,
• be utilized as a tool in therapy; for example, to deliver music therapy,
• have the physical ability to help with tasks requiring dexterity, such as opening medication bottles.

Robots performing monitoring and assisting with medical tasks have been investigated (Breazeal, 2011; Robinson et al., 2014), echoing design recommendations found in this study. With regards to integration, participants focused on *health-based applications* including body signal monitoring and exercise monitoring. Their stories’ support design recommendations for a robot connected to other wearable systems such as a Fitbit or Apple Watch.

**FIGURE 2:**
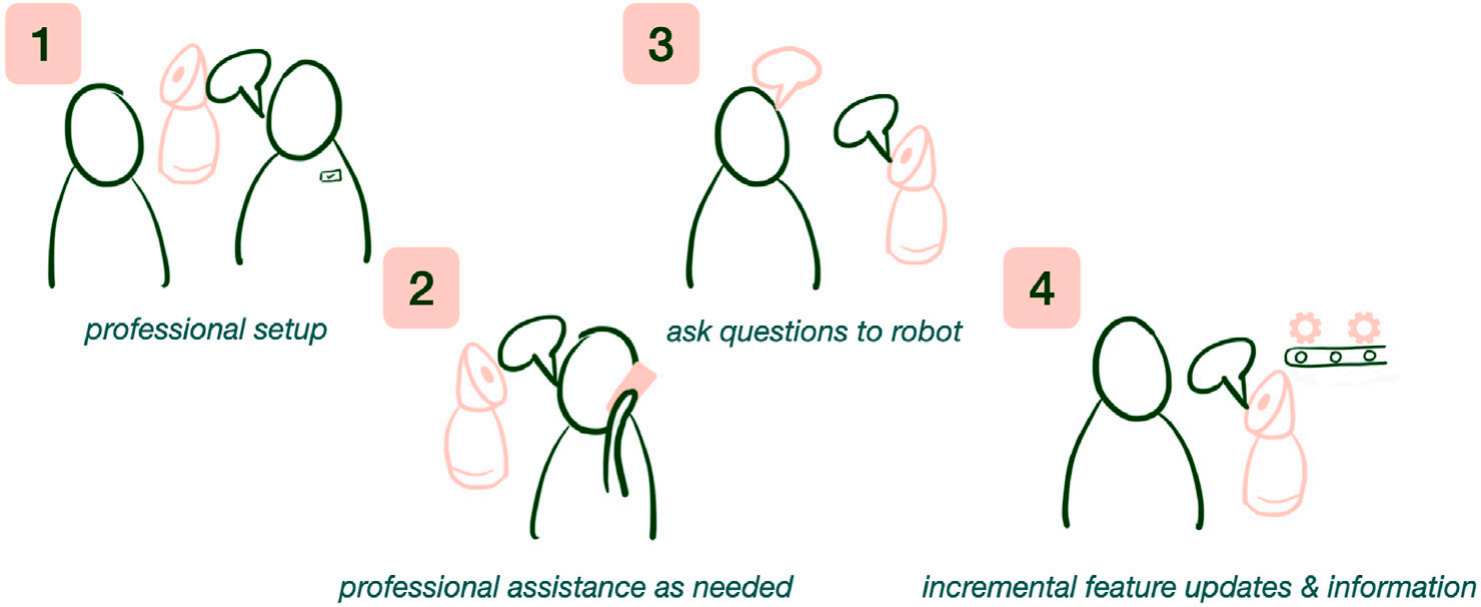
Installation methods recommended by participants including four options: expert setup and assistance, user setup with access to professional assistance, user setup with ability to ask questions to the robot, and robot incrementally introducing new features and supporting older adults using them.

Considering ease-of-use, participants recommended several installation methods that researchers should consider when designing robots that will be introduced into people’s environments without supervision (Figure 2):

• Option 1: Expert setup and assistance with the robot [also recommended by (Lee and Coughlin, 2015)].
• Option 2: User setup that is easy and the user has access to professional assistance as necessary.
• Option 3: User setup with ability to ask questions to the robot to learn how to use the technology.
• Option 4: Robot will incrementally introduce new features and be able to support and encourage older adults to use the technology (Dewar and Dutton, 1986; Green et al., 1995; Ezer et al., 2009).

Researchers could employ a combination of these methods for successful introduction to the technology. Previous studies have found that integration of systems that emphasize simplification are favored and can be better accepted by older adults (Rodríguez et al., 2009; Lee and Coughlin, 2015). Our findings assert that considering seamless setup and on-boarding may promote social robot adoption.

Our work has demonstrated how storytelling can be included in the design of social robots to empower older adults in co-design. We also advocate for storytelling analysis being further incorporated into co-design studies of social robots. Therefore, we provide the following recommendations for how stories can be better elicited and applied to social robot design to extract design recommendations: 1) include open-ended prompts and questions to foster open conversation; 2) in design based activities, provide time for participants to reflect on why they created the design and ask prompts around the social context of the design; and 3) create strong rapport and personal connection with participants, ensuring reciprocity of sharing between participant and researcher. Upon extracting the stories from analysis, researchers can follow a grounded-theory approach (Charmaz, 2014) as in this work to reveal salient themes that can translate to social robot design recommendations.

## 6 Future Work

Participants’ stories also revealed areas where older adults are optimistic of having a social robot. Future research needs to further investigate how older adults’ would like a robot to fit into these contexts: 1) social connection and 2) learning and engagement.

Participants demonstrated openness for a social robot to engage in their social environment and promote human-human interactions. Social robots have been found to provide a sense of social presence and communication that may reduce loneliness and social isolation and enable older adults to connect with family, friends, and their community (Beer and Takayama, 2011; Šabanović et al., 2015; Ostrowski et al., 2019). Further investigation is necessary to understand the balance of creating a device that people see value in using consistently while still promoting and preserving human-human interactions. Stories from this analysis also indicated an interest in robots for *learning and engagement*. Previously, social robots have been shown to promote curiosity and engagement in children (Gordon et al., 2015; Park et al., 2017), and it would be valuable to explore such functionality with older adults. Future work may focus specifically on visualizing stories related to the concepts of learning and social connection.

Lastly, while researchers are delineating the design recommendations for robots in any area of older adults’ lives, they must consider older adults’ dignity of care and autonomy (Sharkey, 2014; Körtner, 2016). These considerations have also been advocated for by participants from this study. As robots are being designed to be involved with older adults’ health, privacy, security and trust must be considered as ethical design considerations in domestic robots (Körtner, 2016). Risks can be mitigated by increasing transparency in data collection, protection of data through legal registration and ethics boards, and emphasizing informed consent from older adults (Körtner, 2016). Future work should be done to promote equitable participatory design of social robots with older adults.

## 7 Conclusion and Study Limitations

Our analysis illustrates the value of considering personal narratives and experiences as heard through storytelling in the process of co-design. Findings of this study indicate that older adults’ stories can be useful to the conceptualization of newer technologies such as social robots and inform several design recommendations for these devices.

We acknowledge that there are a few limitations of our study. First, our sample was comprised largely of individuals recruited from a geographical location in the US where there are many colleges and universities, and individuals are more likely to engage in research. Recruitment from this geographic area may have also contributed to an all-white participant sample. Second, there are some limitations to participatory design as a method including the requirement of large amounts of time, resources, and institutional commitment that are required to complete participatory design projects, and the projects can also require continuous critical commitment by participants which can be a challenge to ensure (Spinuzzi, 2005). In participatory design, researchers must “cede considerable control to their participants and share a ‘design language’ with those participants which must by its nature be imprecise” (Spinuzzi, 2005). While these factors can be limitations, we agree with Wall and Mosher (1994) that the outputs from these co-engagements create artifacts that can be used in multiple ways including “records of a field study; tools for analysis; communication tools for a language game in which researcher-designers and users participate; and focal artifacts for co-design and codevelopment, demonstrating rigor of results.” Third, as we required WiFi access to host a social robot in their home, those who did not have WiFi access could not participate in the study. Previous research acknowledges that access to such resources can further widen the gap between who can engage with technology (Wu et al., 2015; Hargittai et al., 2019), and that there must be ethical considerations in basing technology research on those populations alone (Veinot et al., 2018). Thus, future work should include perspectives from subsets of the aging population that represent various cultural, ethnic, and economic backgrounds, as well as geographic areas.

## Data Availability

The datasets presented in this article are not readily available because the gathered participant transcripts are not shareable. Requests to access the datasets should be directed to akostrow@media.mit.edu.
